# Code Wisely: Risk assessment and mitigation for custom clinical software

**DOI:** 10.1002/acm2.13348

**Published:** 2021-07-10

**Authors:** Rex A. Cardan, Elizabeth L. Covington, Richard A. Popple

**Affiliations:** ^1^ Department of Radiation Oncology University of Alabama at Birmingham Birmingham AL USA

**Keywords:** risk analysis, scripting, software

## Abstract

**Purpose:**

The task of software development has become an increasing part of the medical physicist's role. Many physicists who are untrained in the best practices of software development have begun creating scripts for clinical use. There is an increasing need for guidance for both developers and medical physicists to code wisely in the clinic.

**Materials and Methods:**

We created a novel model for assessing risk for custom clinical software analogous to failure modes and effects analysis and propose minimum best practices that should be followed to mitigate the risks. Using this risk model, we integrated a literature review and institutional experience to form a practical guide for risk mitigation.

**Results:**

Using this new risk assessment model, we outlined several risk mitigation techniques including unit testing, code review, source control, end‐user testing, and commissioning from the literature while sharing our institutional guidelines for evaluating software for risk and implementing these strategies.

**Conclusion:**

We found very little literature for custom software development guidelines targeted at medical physicists. We have shared our institutional experience and guidelines to help facilitate safe software development for the evolving role of the medical physicist.

## 1 INTRODUCTION

In‐house clinical software development has been around for decades but has become more prominent in recent years. Acknowledging the debate whether or not good non‐commercial medical software development management should be considered outside of the scope of medical physics practice,^1^ there is nonetheless the need for the software being actively developed now to be safe and robust for clinical use. Unfortunately, the industry guidelines and staffing models have not changed significantly to accommodate the increased need for quality software development practices in the clinic. At the time of this publication, clinical software development and evaluation are not included in the Commission on Accreditation of Medical Physics Educational Programs (CAMPEP) Standards for Residency Programs.^2^ Physics errors have the potential to impact large numbers of patients because of the scope of responsibilities (eg. machine calibration, planning system commissioning) and lack of qualified personnel to review work and catch errors. In‐house clinical software further amplifies this issue allowing for a complicated task to be automated and scaled throughout a clinical practice potentially causing unintended harm to patients if an error exists in design or implementation.

There has been some guidance published concerning software best practices for medical physicists. More than two decades ago, Rosen^3^ outlined comprehensive practices including writing design specifications, testing, documentation, and other coding practices. Many are still very relevant today, but the software development industry has evolved significantly since that work and we believe there is a need for further practical guidelines. Others have more recently summarized principles they have followed including FMEA and “automated QA”.^4^, ^5^, ^6^ While there have been a few publications on standards for the development of medical device software,^7^ there has been very little with specific targeted recommendations for medical physicists. In the following sections, we will outline a risk assessment model that can be used for clinical software development and highlight some mitigation strategies to give direction that is practical and applicable to medical physicists.

### 1.1 A note concerning spreadsheet software

Many computer applications have the capability for users to modify the behavior of the application or to manipulate data in a user‐specified fashion. When used in this way in a clinical setting, these applications present the same risks as custom software created using a formal programming language. An important example for medical physicists is the spreadsheet program Microsoft Excel (Microsoft Corporation, Redmond, WA). Microsoft Excel is ubiquitous and most medical physicists have experience using it. In a survey of in‐house software practices, Salomons and Kelly^8^ found that 93% of surveyed centers use custom spreadsheets. Clinical physicists who do not consider themselves “programmers” are often comfortable building spreadsheets for clinical use. The familiarity of medical physicists with spreadsheet software can result in complacency about the hazards presented by spreadsheets. Because spreadsheets are so widely used and present unique challenges for quality assurance, risk assessment, and mitigation for spreadsheets will be addressed explicitly along with other in‐house developed software.

## 2 RISK ASSESSMENT

To assess which quality assurance measures are appropriate for the developed software, a risk management approach should be used comparable to failure modes and effect analysis (FMEA).^9^, ^10^ In FMEA, the risk is determined by assessing the frequency of occurrence (O), severity (S), and the probability of the incident going undetected (D) for each identified failure mode. These numbers are multiplied together to obtain a risk priority number (RPN) which is used to quantitatively evaluate the risk of each failure mode in a radiotherapy workflow. Similarly, we recommend that risk should be assessed for each individual software tool developed. Quantitative risk assessment for software can be performed by the following parameters: population (P), intent (I), and complexity (C). Table 1 shows the descriptions of the P, I, and C values.

**TABLE 1:** Descriptions of the P, I, and C values used to assess the risk of custom clinical software

Rank	Population (P)		Intent (I)		Complexity (C)	
	Qualitative	Frequency (%)	Qualitative	Class	Qualitative	Quantitative
1–2	Software used for a specific patient or rare procedure	<1%	No direct clinical impact	I A	Simple	Readable, and <20 lines per unit, and <20 units
3–5	Software used in special procedures	10%	Only impacts clinical efficiency	I B	Somewhat complex	Moderately difficult to read, or 20–50 lines per unit, or 20–50 units
6–8	Software used in routine clinical workflows for every patient	100%	Software used for direct clinical decision making but does not write to a clinical database	II A	More complex	Difficult to read, or 50–100 lines per unit, or 50–100 units
9–10	Software shared across institutions	Multi‐institutional	Software used for direct clinical decision making and writes to a clinical database	II B	Complex	Indecipherable, or >100 lines per unit, or >100 units

Population is intended to gauge the scale of the software tool and is a direct measure of the percentage of the clinic's population that the tool will impact. This is analogous to occurrence in TG‐100 since it a measure of frequency. In effect, it measures the potential amplification of errors in the system. The lowest rank in the category is reserved for software tools that impact a relatively low number of patients, while the highest level includes software tools that will be shared across institutions.

Intent refers to the classification of the software and how it is used in clinical decision making. This is analogous to the severity in TG‐100 because tools that directly impact clinical decision making pose a larger risk than those used for efficiency improvements. We stratified classes by their ability to acutely impact patient outcomes and whether they could override existing clinical data. A class system similar to how the FDA determines risk for medical devices^11^ is shown in Table 2 but has been tailored to software categories expected to be developed in medical physics.

**TABLE 2:** Classification system for the intent of clinical software

Classification	Description	Examples
I A (Minimal risk)	A codebase that does not have a direct impact on clinical efficiency or clinical outcome (research)	Plan automation in research database; DVH Mining; Post‐treatment plan analysis
I B (Minimal to moderate risk)	A codebase that could influence clinical efficiency but not directly affect the clinical outcome if code does not perform as anticipated or if the design is flawed (efficiency tools)	DICOM automation; Export tools; Post‐treatment Reporting
II A (Moderate Risk)	A codebase which could affect the clinical outcome if code does not perform as anticipated or if the design is flawed but does not write to a clinical database (read clinical tools)	Plan quality check tools; Pre‐treatment reporting/instructions
II B (Moderate to High Risk)	A codebase which could affect the clinical outcome if code does not perform as anticipated or if the design is flawed and also writes to a clinical database (write clinical tools)	Plan automation components; QA automation components; Pre‐treatment data importing tools

Risk is also increased as the complexity of the code increases. According to Leveson, one of the most effective tools for making software safer is building it to be intellectually manageable.^12^ Complexity is a measure of how difficult it is to find an error by an independent reviewer. This is analogous to TG‐100 detectability. With increased complexity, the probability of errors going undetected significantly increases. We broke down complexity into three main categories: number of lines of code per unit/function, the total number of units, and readability of the code. Readability is a qualitative measure that includes following some standard practices of variable naming, method naming, and sufficient comments so that the intent of the methods and variables can be easily understood. Low complexity improves the maintainability of the code base and prevents increased risk when the original developer(s) are no longer a part of the project.

Like RPN, these parameters can be used to rank each custom software tool by multiplying the parameters together to get the software risk number (SRN) where SRN = P*I*C. This number will serve as a quantitative metric for the posed risk if the code does not perform as anticipated or if the design is flawed. After SRN is evaluated, clinics can rank tools by highest SRN and intent to determine the most hazardous tools. Tools with the highest SRN and those used for direct clinical decision making (IIA, IIB) can be allotted the appropriate resources during development, commissioning, and routine quality assurance. Our intent is not to be prescriptive in what SRN values require which isk management tools, but to create a framework for assessing risk. Individual clinics can evaluate SRN in conjunction with available resources to prioritize and allot risk management endeavors.

### 2.1 Spreadsheet software

For the risk assessment of spreadsheets, the population and intent rankings can be assigned as described above. For complexity, a separate evaluation is needed. Table 3 shows the complexity ranking for spreadsheets. Complexity increases as the number of cells and the interdependence of calculated cells increases. The highest level of complexity is for spreadsheets that contain macros regardless of the number of calculated cells.

**TABLE 3:** Tiers of complexity for spreadsheets

Rank	# Calculated cells	# Sheets with data input	Macros
1–2	<10	1	No
3–5	<25	1	No
6–8	>25	2+	No
9–10	‐	‐	Yes

### 2.2 Reassessment of risk

**FIGURE 1:**
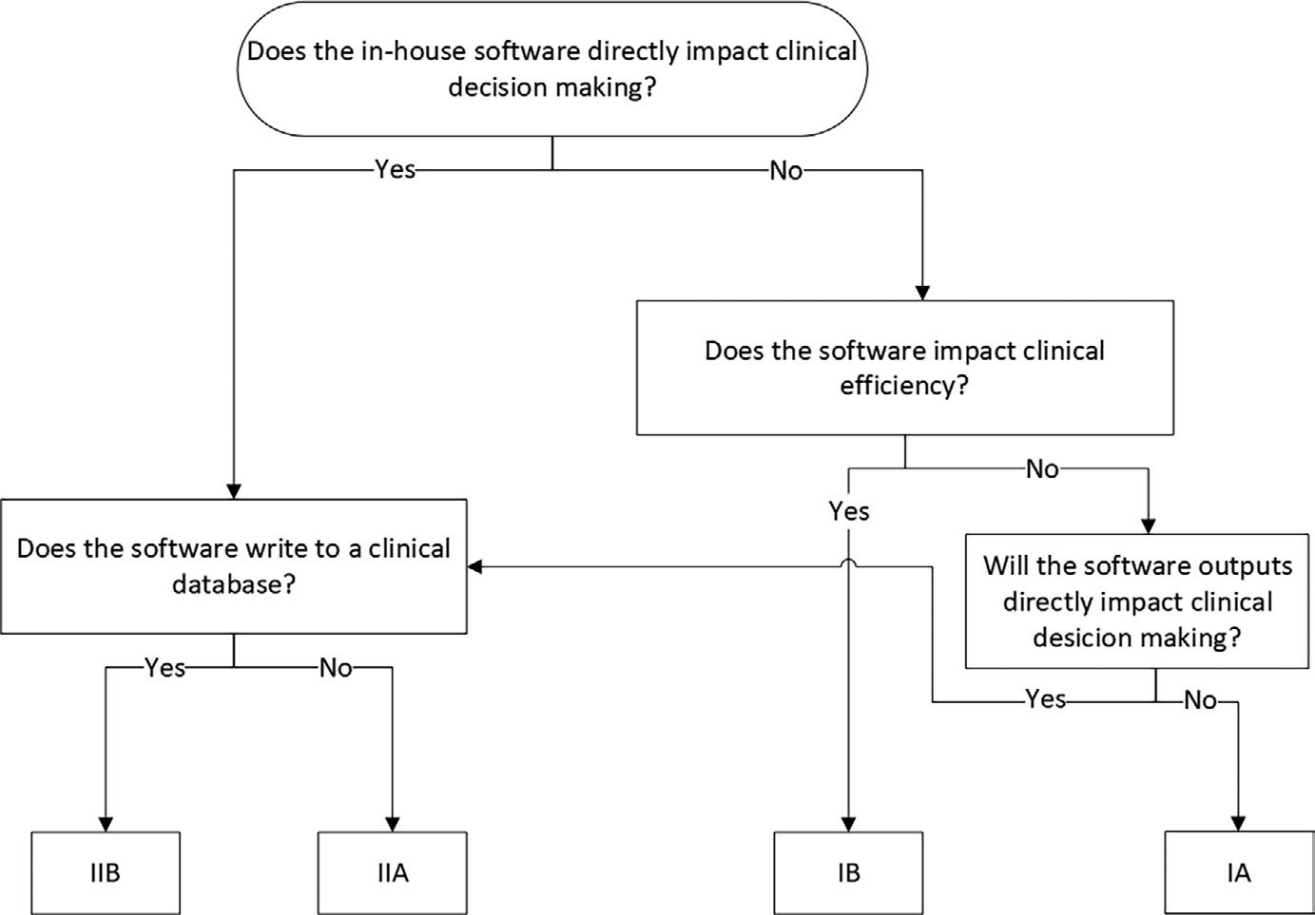
Decision tree for software classification

It should be noted that risk can change as the project progresses. For example, while a large outcome project would have no immediate clinical impact (categorized as a Class IA initially) and a low population score (Rank 1), its errors could have a long‐lasting impact if the data were eventually used for setting clinical objectives. In this case, the code could transition to a population Rank 3 or 4 and intent of II A, as shown in Figure 1, increasing the risk significantly. We recommend regularly assessing the risk of the software throughout the project timeline to ensure that adequate risk mitigation techniques have been employed.

### 2.3 Risk and the end‐user

We note that this model does not explicitly account for the end‐user when classifying detectability and complexity. We acknowledge that the detectability of errors can vary depending on the user and are dependent on both their technical expertise and familiarity with the software content. Complexity levels could also vary as the readability of the program is subjective. When assessing detectability and complexity, it may be prudent to consider the characteristics of the end‐users and adjust these parameters accordingly.

## 3 RISK MITIGATION

According to Leveson, “complacency is the most important risk factor in a system, and a safety culture must be established that minimizes it.”^12^ Within the context of an established safety culture, best practices of software development include a diverse team to develop and evaluate the software. Table 4 includes the roles of responsibilities of team members. Typically, physicists are serving multiple roles on the teams as both developers and users. If feasible, physicists not involved in direct code development should be available for end‐user testing and commissioning. We acknowledge that this may not be possible due to staffing at small clinics. Regardless, end‐user testing and commissioning should be well documented to distinguish development from quality assurance steps.

**TABLE 4:** Role and responsibilities for the development of in‐house software

Role	Responsibilities
Developer	Source control
	Unit testing
	Integration testing
	Code documentation
Physicist	Risk assessment
	End‐user testing
	Commissioning
	Policies and procedures
User	End‐user testing
	Product improvement requests
	Bug reporting

### 3.1 Software configuration

When possible, editing of configuration parameters should be controlled to eliminate the risk of unintended changes. This is difficult to achieve at run‐time because it requires the creation and management of user roles. If configuration parameters cannot be secured at run‐time, developers should strongly consider incorporating parameters into the application so that when compiled the parameters are not exposed to end‐users. Alternatively, configuration files can be placed in a location that users cannot access, or the configuration files can be encrypted and decrypted by the application. For spreadsheets, the entire document is like a configuration file and careful consideration should be taken to ensure critical parameters and functions do not change unintendedly. Specific recommendations for spreadsheets are outlined below.

### 3.2 Source control

One of the most difficult tasks about malleable custom software is knowing its current state. Has it been modified? Who modified it? Why was it modified? Is the current version tested? Which version is deployed? Software configuration management (SCM) aims to solve these problems by allowing a small database to keep track of every detail of changes over time. The authors believe this is one of the most crucial steps needed to implement a safe and clinical development practice. Modern source control systems, most popularly Git,^14^ allow many other advantages including allowing easy editing on multiple computers by multiple people.

### 3.3 Code review

When possible, higher risk software should be reviewed by an independent party. McIntosh et al. found that “review coverage, participation, and expertise share a significant link with software quality.”^15^ Code review helps enforce good practices of naming, encapsulation, and overall readability which reduces the complexity of code and thereby increasing detectability. The extent and detail of the review are subjective. At our institution, we review all Class II software (Intent) and ensure the code is readable, logical, and maintainable.

### 3.4 Testing

Testing software can be a tedious task. Testing is often divided into several categories including function (unit) testing, system testing, volume testing, capacity testing, security testing, performance testing, and many others.^16^ Because the scope of testing can be overwhelming for a clinical medical physicist, we have chosen to describe the minimum testing which should be performed prior to deploying clinical software. We believe the following are achievable and practical techniques that are followed at our institution for Rank 3 and above (Population) and Class IB and above (Intent).

#### 3.4.1 Unit testing

As highlighted by Levenson,^17^ specification of software and isolation of safety‐critical components is paramount to safe software practices. One practical way of both specifying the objective and isolating a part of the software from other parts is to design the entire application as a collection of smaller units. This allows for the common practice of developers to strengthen a code base with unit testing, or “testing the smallest separate module in the system.”^18^ Unit testing is like component testing (machine QA) for medical physicists, where the entire validation of a medical device is broken into subtests (rotation about isocenter, mechanical motion along each axis, energy validation, etc.). In modern unit testing frameworks, the tests are automated and can be run upon any change to the code. Deciding which units should be tested in this way is subjective, but we recommend at a minimum to design unit tests for the most critical parts of code. Criticality can be assessed using the risk model outlined previously.

#### 3.4.2 System testing

System testing is the overall testing of a complete system, a concept familiar to medical physicists as end‐to‐end testing. Leveson states that the “safety of software can only be evaluated in the context of the system within which it operates.”^12^ System tests should be defined early in the development process, including the expected output of each test. System integration testing should not be deferred to the end but should start as soon as possible during development. All system tests should be completed successfully prior to each round of end‐user testing.

#### 3.4.3 End‐user testing

In a recent survey on the development of in‐house software by medical physics,^8^ testing was reported as the area of most concern when developing software for clinical use. In addition to the testing done by developers, end‐user testing, or user acceptance testing is necessary before the clinical release of software tools. End‐user testing will be used to test the system for unforeseen use cases that can result in errors. One of the most infamous instances of this in radiotherapy was the Therac‐25 software which could trigger catastrophic radiation doses in patients if the therapists pressed the console buttons too quickly.^19^ Before end‐user testing, a testing plan should be developed. This plan should detail to the end‐user a list of tests with specifications. Per Rosen^3^ tests should be based on risk analysis. End users should document which tests were performed with outcomes to either provide feedback to the development or to document functionality for commissioning reports.

Per Padmini et al.,^20^ end‐user testing should be done early and throughout the development period to prevent project failure. To optimize end‐user testing, developers should provide the end‐users with documentation and clear instruction on how the software operates. End users cannot expect to test software without an understanding of the functionality of the program. Padmini et al. identified the lack of a proper end‐user testing process and lack of knowledge of the testing procedure as common shortcomings in end‐user testing. Per Salomons et al.,^8^ a survey indicated that only 7% of in‐house software tools had written guidelines for the use of in‐house software. This shows an area of need for great improvement to follow best practices in software development.

### 3.5 Clinical commissioning

Before clinical release, in‐house software should undergo a formal commissioning process to assess the software for clinical release. This is independent of software development testing but may include an overlap of tests performed during end‐user testing. Tests performed should be clearly documented and include test plans or anonymized patients used for evaluation. Outcomes of commissioning should include a formal commissioning report, use policy, and procedures for users.

#### 3.5.1 Spreadsheets

One resource for spreadsheet development is The European Spreadsheet Risks Interest Group (EuSpRig) which maintains a list of references for best practices and a collection of spreadsheet horror stories.^21^ While many of the practices EuSpRig focus on the financial industry, concepts such as documentation and team review are generalizable to any field. Because spreadsheets are rarely built by professional developers and spreadsheet software was not designed for life safety applications, industry‐standard tools for testing and deployment, specifically unit testing tools, are not readily available and well known although commercial software for version control and unit testing of Excel is available. Many of the practices described here can be readily applied including code classification, version control, integration testing, and clinical commissioning. In addition to these practices, several spreadsheet‐specific strategies should be applied. First, cells that do calculations or that contain fixed data should be protected. In our experience, clinical users will not hesitate to unlock cells to fix a perceived problem and so protecting cells should be augmented with a password requirement to unlock cells. Second, spreadsheets should be developed as templates that prevent editing by making the files read‐only. Completed spreadsheets should not be copied and pasted to be used with new data. In addition to the risk of using old data, copying spreadsheets, rather than using a template, can result in multiple versions of a spreadsheet being in use. If spreadsheets are not appropriately protected, copying files can result in user‐introduced errors being distributed.

## 4 CONCLUSION

Custom clinical software development is increasing in medical physics and there is little published guidance to help physicists in both the assessment of risk and techniques to reduce risk. We feel many of the methods mentioned are beyond the typical expertise of a physicist and needed to be elucidated. We have outlined a novel strategy for categorizing clinical software to determine the prioritization of effort to afford to create a safe software development practice. These guidelines can be applied to a variety of custom clinical software from stand‐alone programs, spreadsheets, and application programming interface (API) scripts for treatment planning systems and other commercial software that allows APIs. Additionally, we have shared some of our institution's thresholds and techniques which we believe to be practical and achievable at most facilities.

## CONFLICT OF INTEREST

The authors have no relevant conflicts of interest to disclose.

## AUTHOR CONTRIBUTION

Rex A. Cardan made substantial contributions to the conception and design of the work; the acquisition, analysis, and interpretation of data for the work; drafting the work and revising it critically for important intellectual content; gave final approval of the version to be published; and agrees to be accountable for all aspects of the work in ensuring that questions related to the accuracy or integrity of any part of the work are appropriately investigated and resolved. Elizabeth L. Covington made substantial contributions to the conception and design of the work; the acquisition, analysis, and interpretation of data for the work; drafting the work and revising it critically for important intellectual content; gave final approval of the version to be published; and agrees to be accountable for all aspects of the work in ensuring that questions related to the accuracy or integrity of any part of the work are appropriately investigated and resolved. Richard Popple made substantial contributions to the conception and design of the work; the acquisition, analysis, and interpretation of data for the work; drafting the work and revising it critically for important intellectual content; gave final approval of the version to be published; and agrees to be accountable for all aspects of the work in ensuring that questions related to the accuracy or integrity of any part of the work are appropriately investigated and resolved.

## Supporting information

Supplementary Material
