# Supplementary material for: Code Wisely: Risk assessment and mitigation for custom clinical software
Source: J Appl Clin Med Phys. 2021 Jul 10;22(8):273–9. doi: 10.1002/acm2.13348 (PMC8364260; doi:10.1002/acm2.13348)
Supplement: Supplementary file 1 — Supplementary Material [file ACM2-22-273-s001.docx]

**Code Wisely: Risk assessment and mitigation for custom clinical software**

Running title: Risk assessment for custom clinical software

Rex A. Cardan, Elizabeth L. Covington, Richard A. Popple

Department of Radiation Oncology

University of Alabama at Birmingham, Birmingham, AL

1719 6th Ave S, Birmingham, AL 35294

Corresponding Author:

Rex Cardan, PhD

[rcardan@uabmc.edu](mailto:rcardan@uabmc.edu)

(205) 975-1857

Author Contribution Statement

Rex A. Cardan made substantial contributions to the conception and design of the work; the acquisition, analysis, and interpretation of data for the work; drafting the work and revising it critically for important intellectual content; gave final approval of the version to be published; and agrees to be accountable for all aspects of the work in ensuring that questions related to the accuracy or integrity of any part of the work are appropriately investigated and resolved.

Elizabeth L. Covington made substantial contributions to the conception and design of the work; the acquisition, analysis, and interpretation of data for the work; drafting the work and revising it critically for important intellectual content; gave final approval of the version to be published; and agrees to be accountable for all aspects of the work in ensuring that questions related to the accuracy or integrity of any part of the work are appropriately investigated and resolved.

Richard Popple made substantial contributions to the conception and design of the work; the acquisition, analysis, and interpretation of data for the work; drafting the work and revising it critically for important intellectual content; gave final approval of the version to be published; and agrees to be accountable for all aspects of the work in ensuring that questions related to the accuracy or integrity of any part of the work are appropriately investigated and resolved.
